# Current trends in waste valorization

**DOI:** 10.1111/1751-7915.14198

**Published:** 2022-12-21

**Authors:** Eldon R. Rene, Prakash K. Sarangi, Violeta Sànchez i Nogué, Anna Schnürer, Davinia Salvachúa

**Affiliations:** ^1^ Department of Water Supply, Sanitation and Environmental Engineering IHE Delft Institute for Water Education Delft The Netherlands; ^2^ College of Agriculture Central Agricultural University Imphal India; ^3^ Renewable Resources and Enabling Sciences Center National Renewable Energy Laboratory Golden Colorado USA; ^4^ Department of Molecular Sciences Swedish University of Agricultural Sciences Uppsala Sweden

## Abstract

This paper presents the scientific breakthroughs made in bioprocess engineering and microbial biotechnology for the conversion of wastes into products with added value and/or biofuels. The significant results obtained in the emerging fields of hybrid electrosynthesis, the role of enzymes in the degradation of plastics, polyhydroxyalkanoate and 5‐aminolevulinic acid production, fermentation technology and the application of molecular engineering tools to bioprocess technology are highlighted.

## Abstract 2

This paper presents the scientific breakthroughs made in the fields of bioprocess engineering and microbial biotechnology. The article focuses on the conversion of waste to value‐added products and biofuels.

## INTRODUCTION

Organic solid waste (e.g. food waste, sludge and residues, agricultural wastes) is an underutilized feedstock for the production of biofuels, biochemicals and biomaterials. The valorization of these wastes to value‐added products remains difficult due to substrate heterogeneity and recalcitrance, low carbon conversion efficiencies to achieve specific, profitable products during biological conversion, and the complexity of downstream and scale‐up processes. Anaerobic digestion systems are excellent examples of waste valorization and are now the basis for research aimed at generating products from waste (beyond biogas) in order to achieve a circular (bio)economy. This special issue was aimed at covering the following aspects:

- Waste conversion by isolated microorganisms and microbial consortiums
- Microbial systems and synthetic biology
- Bioprocess development and process engineering for waste valorization
- Techno‐economic analyses and life cycle assessments of bioconversion processes

## TECHNOLOGIES FOR WASTE VALORIZATION

Waste valorization, also referred as resource recovery from waste, is the process of reusing, recycling or processing waste materials and transforming them into value‐added products such as platform chemicals, fuels and bioenergy. Sravan and Mohan (2022) provided valuable ideas for waste valorization within the framework of circular economy by emphasizing the role of hybrid electrosynthesis as a non‐genetic method for regulating microbial metabolism. The authors noted that waste‐fed biorefineries might be a technologically and economically viable benchmarking platform with customizable technology that could be adapted to the characteristics of solid waste, wastewater and biomass. The authors clearly presented the difficulties associated with the long‐term stability of enzymes, cofactors and metabolites; the optimization of electrochemical/material needs; and the process's techno‐economics. Thus, it was emphasized that current research on the role of novel materials and their role in electron transfer during biological processes with diverse microenvironments and electrochemical interventions must be examined after identifying the input and analysing their physical, chemical and biological characteristics.

## DEGRADATION OF PLASTICS

Plastics have widespread application in fields as diverse as electronics, medicine, agriculture, consumer products and chemical industries. The disposal and accumulation of plastics in the environment has become a huge global problem due to the fact that these materials are resistant to the natural processes of biodegradation. For example, the rate and kinetics of plastic degradation in the natural environment depends on its chemical structure, molecular weight and crystalline properties. Chow et al. (2022) reviewed and explored the issue of plastic pollution and the involvement/role of microorganisms. The authors centred their review and analysis on the following key points: (i) the physical, mechanical and chemical degradation (weathering) of plastics, microbial plastic colonization, (ii) the role of additional C sources for microbial degradation of plastics, (iii) the common structural traits of polyethylene terephthalate (PET)‐active enzymes, (iv) the model for enzymatic degradation of polyethylene terephthalate (PET) by mixed and multispecies microbial consortia and (v) the future challenges in this field. In this special issue, another work (Xu et al., 2022) has also examined the role of microorganisms in plastic degradation, and its authors have proven the use of a novel fluorogenic probe and fluorescence‐activated droplet sorting (FADS) pipeline for screening polyester‐degrading microorganisms. The authors assessed the specificity and sensitivity of the fluorogenic probe FPAP (fluorescent polyurethane analogue probe) for fluorescence detection in picolitre droplets. The following key findings were emphasized by the authors: (i) when each microdroplet passed through the device, the fluorescence was detected by a photomultiplier tube (PMT), (ii) the microdroplets with higher fluorescence accounted for 95% of the total sorted droplets and (iii) the probe was able to distinguish the leaf‐branch compost cutinase (LCC) polyesterase enzyme.

## POLYHYDROXYALKANOATE (PHA) PRODUCTION FROM WASTES

In light of the global requirement to transition from a fossil fuel‐based bioeconomy to a renewable carbon‐based bioeconomy, research on the manufacturing of plastics from non‐conventional substrates should be conducted. Gutschmann, Högl, et al. (2022) created a pneumatic feeding system for solid fat/protein‐emulsions, that is, converting animal by‐products to polyhydroxyalkanoate using the *R. eutropha* Re2058/pCB113 strain. The authors emphasized the significance of two strategies for process scaling up: (i) converting waste animal fats (WAF) to biodiesel and using the glycerol and saturated fatty acid methyl ester phases for PHA production; and (ii) thermal liquefaction of WAF and direct feeding into an existing emulsion formed with plant oil. Concerning the experiments, the authors tested both shake flask and bioreactor cultivations, and fat/protein emulsion (FPE) was used as the carbon and nitrogen source. In the bioreactor system, a unique feeding mechanism was created to feed the fat/protein‐emulsions, and the results demonstrated a maximum value of 35.8 g/L P(HB‐co‐HHx) and a space–time yield of 0.6 gPHA/L/h.

Gutschmann, Maldonado Simões, et al. (2022) reported the bioconversion of waste animal fat (WAF) to polyhydroxyalkanoates (PHAs) in laboratory scale reactors utilizing *Ralstonia eutropha* Re2058/pCB113, obtaining 45 gPHA/L a space–time yield of 0.63 gPHA/L/h. Surprisingly, minimal PHA accumulation (31.5 gPHA L^−1^) was reported in a 150‐L pilot scale reactor equipped with three 6‐blade Rushton impellers, likely due to the following factors: (i) insufficient or variable WAF, (ii) scale‐up effects and (iii) dense foam formation. Acuña and Poblete‐Castro (2022) evaluated rational engineering approaches for the synthesis and recovery of PHA from various substrates and emphasized the significance of PHA‐producing microorganisms. The authors analysed the fundamentals of PHA metabolism and key precursors, the engineering strategies to increase PHA synthesis from glycerol, methane, methanol and waste streams, the engineering strategies to increase PHA accumulation in CO_2_ using microbes; the cofactor and morphology engineering strategies; and the mechanism of programmable cell lysis and PHA recovery using examples from the literature.

## OTHER VALUE‐ADDED PRODUCTS FROM WASTES

An alternative to the resources that are obtained from fossil fuels is feedstock that is based on biomass, and studies have shown that these biomass‐based feedstocks offer the same benefits for the production of energy or other value‐added products. The common examples of biomass derived wastes include agricultural waste, forest residue, municipal solid waste, food waste and waste from livestock rendering facilities. In this special issue, agro‐waste (green tea residues, GTR) was converted into bioactive peptides via anaerobic digestion, assisted by probiotics (Lee et al., 2022). The authors used the following strains: (i) probiotic strains, (ii) *Lactiplantibacillus plantarum* APsulloc 331261 and (iii) mixed bacterial culture, in anaerobic serum vials sealed with butyl‐rubber stoppers at 37°C, under a gas mixture comprising of 80% nitrogen, 10% carbon monoxide and 10% hydrogen. The following is a summary of the important findings of that study: (a) GTR hydrolysates contained a wide range of lower molecular weight peptide fractions, (b) *Lactiplantibacillus plantarum* appeared to be the most significant producer of GTR hydrolysates with peptides as the primary products, (c) Lactic acid bacteria (LAB) species have different GTR hydrolytic capacities due to the different protease repertoires, (d) GT peptides appeared to be glycosylated primarily by mannose, *N*‐acetyl glucosamine, and galactose and (e) anaerobic digestion valorized GTR hydrolysates into health‐promoting agents, with applications in the nutraceutical and cosmeceutical sectors.

Zhang, Wang, and Chen (2022) conducted a study on the treatment of greenhouse gases in which they monitored the transcriptome profile of *Scenedesmus obliquus* HTB1 across 2, 12, 24 h and 4 days during microalgal adaptation to a 10% CO_2_ concentration. By monitoring the growth, photosynthesis, carbon metabolism, nitrogen metabolism, the cell cycle and signal transduction, the authors reported the changes observed at the transcriptional level. The accelerated cell cycle and MAPK (Mitogen‐activated protein kinase) signalling pathway were discovered to be responsible for faster microalgal growth, while the improved oxidative phosphorylation and P‐ATPases were primarily responsible for pH homeostasis (Zhang, Wang, & Chen, 2022). Thus, according to the authors, carbon metabolism, including fatty acid biosynthesis and the TCA cycle, was significantly improved at 10% CO_2_, making *Scenedesmus obliquus* HTB1 a suitable choice for absorbing CO_2_ from flue gases.

Vyas et al. (2022) offered clear insights into hydrophobic waste valorization for the synthesis of oleochemicals with added value and provided the following recommendations: (i) efficient development of downstream processing is a crucial aspect of commercializing oleochemicals, (ii) marine and salt‐tolerant microorganisms such as thraustochytrids have gained significant attention as promising multi‐product single‐cell biorefineries, and these biocatalysts should be tested on a large scale, (iii) hydrophobic wastes (e.g. waste cooking oils) should be treated properly and used to produce value‐added products and realize circular bioeconomy and (iv) understanding the metabolic pathways and identifying the major enzymes and regulatory factors involved in the valorization of wastes requires an omics‐based approach (transcriptomics, proteomics and metabolomics). 5‐Aminolevulinic acid (ALA) has considerable agricultural applications as a plant growth regulator and as a photosynthesis enhancer (Luo et al., 2022). The hydrolysates of cassava residue (CR) and fish waste (FW) were utilized to manufacture ALA with *Bacillus cereus* PT1 (Luo et al., 2022), and the following findings were reported: (i) using CR hydrolysate (CRH) as a carbon source and tryptone as a nitrogen source, the ALA production was 140 mg/L, (ii) with glucose as a carbon source, FW hydrolysate (FWH) contributed to an ALA production of 195 mg/L and (iii) the ALA production was 2.6 g/L in a 7‐L fermenter employing CRH and FWH. Zhang, Wang, Usman, et al. (2022) investigated the possibility of producing biofuel from legume residues through the enzymatic saccharification of *Caragana korshinskii* Kom., silage treated with a rapid start‐up *Pediococcus acidilactici* strain and *Acremonium* cellulase. They also reported the fermentation profile, structural carbohydrates degradation, enzymatic saccharification and the dynamics of the bacterial community of *C. korshinskii* silage. After 60 days of testing, the authors reported that the cellulose conversion of ensiled *C. korshinskii* was affected by additives and hydrolysis time, with high concentrations of lactic and acetic acids and decreased NPN and NH_3_‐N concentrations. Park et al. (2022) engineered *Halomonas bluephagenesis* TD1.0 to create the biofuel propane, the bioplastic poly‐3‐hydroxybutyrate (PHB), and the biochemicals mandelate and hydroxymandelate, respectively, in a single, semi‐continuous batch fermentation system under non‐sterile conditions. In addition, the authors applied strain engineering to increase (hydroxy)mandelate production by eliminating the genes responsible for tyrosine breakdown. However, the authors also noted that the isolation of high‐value, high‐titre compounds is crucial to achieve economic viability through microbial bioproduction systems.

## CONCLUSIONS

The valorization of waste into value‐added chemicals and bioenergy contributes to the development of a low‐carbon society and promotes a circular bioeconomy. Biofuel, bioplastics, carotenoids, squalene and long‐chain polyunsaturated fatty acids such as docosahexaenoic acid, eicosapentaenoic acid and docosapentaenoic acid can be produced by utilizing various biotechnologies and biocatalysts, as demonstrated by the articles published in this special issue. The application of innovative biotechnologies and engineered microbes for waste valorization has been shown to increase the extraction efficiency, reduce energy consumption and improve the climate by lowering greenhouse gas emissions.

## AUTHOR CONTRIBUTIONS

**Eldon R. Rene:** Conceptualization (equal); formal analysis (equal); writing – original draft (equal); writing – review and editing (equal). **Prakash K. Sarangi:** Conceptualization (equal); formal analysis (equal); writing – original draft (equal); writing – review and editing (equal). **Violeta Sànchez i Nogué:** Conceptualization (equal); formal analysis (equal); writing – original draft (equal); writing – review and editing (equal). **Anna Schnürer:** Conceptualization (equal); formal analysis (equal); writing – original draft (equal); writing – review and editing (equal). **Davinia Salvachúa:** Conceptualization (equal); formal analysis (equal); writing – original draft (equal); writing – review and editing (equal).

## FUNDING INFORMATION

No funding information provided.

## CONFLICT OF INTEREST

There are no conflicts of interest to declare.
